# Plant endophytes promote growth and alleviate salt stress in *Arabidopsis thaliana*

**DOI:** 10.1038/s41598-020-69713-5

**Published:** 2020-07-29

**Authors:** Di Fan, Sowmyalakshmi Subramanian, Donald L. Smith

**Affiliations:** Department of Plant Science, McGill University, Macdonald Campus, 21111 Lakeshore Road, Sainte-Anne-de-Bellevue, Québec, H9X 3V9 Canada

**Keywords:** Plant ecology, Climate change

## Abstract

Plant growth promoting rhizobacteria (PGPR) are a functionally diverse group of microbes having immense potential as biostimulants and stress alleviators. Their exploitation in agro-ecosystems as an eco-friendly and cost-effective alternative to traditional chemical inputs may positively affect agricultural productivity and environmental sustainability. The present study describes selected rhizobacteria, from a range of origins, having plant growth promoting potential under controlled conditions. A total of 98 isolates (ectophytic or endophytic) from various crop and uncultivated plants were screened, out of which four endophytes (n, L, K and Y) from *Phalaris arundinacea*, *Solanum dulcamara*, *Scorzoneroides autumnalis*, and *Glycine max*, respectively, were selected in vitro for their vegetative growth stimulating effects on *Arabidopsis thaliana* Col-0 seedlings with regard to leaf surface area and shoot fresh weight. A 16S rRNA gene sequencing analysis of the strains indicated that these isolates belong to the genera *Pseudomonas*, *Bacillus*, *Mucilaginibacter* and *Rhizobium*. Strains were then further tested for their effects on abiotic stress alleviation under both Petri-plate and pot conditions. Results from Petri-dish assay indicated strains L, K and Y alleviated salt stress in *Arabidopsis* seedlings, while strains K and Y conferred increases in fresh weight and leaf area under osmotic stress. Results from subsequent in vivo trials indicated all the isolates, especially strains L, K and Y, distinctly increased *A. thaliana* growth under both normal and high salinity conditions, as compared to control plants. The activity of antioxidant enzymes (ascorbate peroxidase, catalase and peroxidase), proline content and total antioxidative capacity also differed in the inoculated *A. thaliana* plants. Furthermore, a study on spatial distribution of the four strains, using either conventional Petri-plate counts or GFP-tagged bacteria, indicated that all four strains were able to colonize the endosphere of *A. thaliana* root tissue. Thus, the study revealed that the four selected rhizobacteria are good candidates to be explored as plant growth stimulators, which also possess salt stress mitigating property, partially by regulating osmolytes and antioxidant enzymes. Moreover, the study is the first report of *Scorzoneroides autumnalis* (fall dandelion) and *Solanum dulcamara* (bittersweet) associated endophytes with PGP effects.

## Introduction

Many studies have reported that microbes associated with root systems are generally abundant and manifest beneficial activity as plant growth promoting rhizobacteria (PGPR) by stimulating plant growth, reducing pathogensis, and allievating abiotic stresses, through a set of underlying mechanisms; this understanding has been widely examined^1^. These PGPR are part of the regulated community of microbes, the phytomicrobiome that is associated with each plant^2,3^. This community plus the plant forms the holobiont, the entity that evolution acts upon and that generates biomass and food in agricultural systems^4^. In order to provide adequate food and nutrition worldwide, agricultural production faces challenges, in which chemical fertilizers as well as pesticides have become one of the limiting factors due to their increasing costs, limited availability and negative impact on agro-ecosystems^5^. Moreover, increasing desire for food, free of chemical residues, has boosted demand for biofertilizers as a sustainable option for improving plant growth and yields in an environmentally friendly manner^6^. Use of microbial consortia for reducing chemical inputs without compromising yield is now an important feature of research in agriculture, microbiology and biotechnology^7^. Thus, the screening and identification of PGPRs has gained considerable attention. Strains of root associated bacterial genera including *Azospirillum*, *Arthrobacter*, *Azotobacter*, *Bacillus*, *Burkholderia*, *Erwinia*, *Enterobacter*, *Klebsiella*, *Paenibacillus*, *Pantoea*, *Pseudomonas*, *Serratia*, and *Xanthomonas* are among the main rhizobacteria investigated for promotion of plant growth^8–10^; some of these have already been deployed as biofertilizers^11^.

The fossil record shows that mycorrhizal interactions with plants have occurred for more than 400 million years^12^; this relationship has been studied extensively and molecular biology and ‘omics’ have provided approaches to the mechanisms, regarding the beneficial physiological and molecular interactions^13^. Likewise, interactions between plants and nitrogen-fixing bacteria, especially legumes and rhizobia have been very well investigated^14,15^. Further, various beneficial interactions between plants and PGPR (those that colonize either the rhizosphere, rhizoplane or within plant root tissues) are involved in a wide range of PGP activities^16,17^, such as nutrient solubilization, N_2_ fixation, production of siderophores, volatile compounds and plant-like hormones, induction of systemic resistance, as well as direct biocontrol of disease organisms^8,18^. Recently, some additional PGP traits have been discovered, such as bacteriocin production, microbe-to-plant signals^19^ and sulfur deficienciy alleviation^20^.

Plants are multi-cellular organisms that cope with various environmental stresses, the most common being soil salinity, cold temperature and drought, all of which can elicit some common gene responses related to nearly every aspect of plant morphology, physiology and metabolism^21,22^, leading to inhibition of seed germination, seedling growth, flowering and seed set^23^. Soil salinity adversely affects plant growth via both osmotic and ionic stresses and has become a major limiting factor in agricultural production worldwide, leading to as much as $27 billion USD in losses per year^24^. Mechanistically, high salinity results in high concentrations of sodium (Na^+^) and chlorine (Cl^−^) ions and low potassium (K^+^) levels, after prolonged exposure^25,26^. Salinity stress also disrupts the cellular osmotic balance by lowering the water potential inside the cells^27^. Stressfully high salt conditions induce oxidative stresses by generating reactive oxygen species (ROS)^28^ within cells, resulting in oxidative damage of membrane lipids, proteins and nucleic acids^29^. To cope with salt stress, stressed plants activate various mechanisms through conserved signal transduction pathways^26^, resulting in the production and accumulation of diverse functional components such as osmolytes (i.e., proline and glycine betaine^30^) and non-enzymatic (i.e., phenolics, flavonoids, and glutathione) and enzymatic antioxidants (i.e., peroxidase, catalase, as well as the enzymes involved in ascorbate–glutathione cycle)^31^, all of which mitigate the oxidative damage caused by high salinity^26,32^. A promising alternate to conventional breeding and genetic transformation, to develop salt stress tolerant plants, is to introduce PGPR into plants under salinity stress.

There is a clear need for increased crop plant production due to a growing world population, diminishing tillable land area and increasing meat consumption, all of which are driving the search for novel microbes to develop effective bioinoculants. Bioinoculants with characteristics for plant growth promotion and salt stress resistance, can be added to both crop plants and biomass-producing plants to underpin the growing bioeconomy and allowing production of advanced biofuels and associated high-value bioproducts, e.g. *Panicum virgatum* (switchgrass)^33^. Chemical fertilizers cause large increases in crop production but are expensive and can cause severe environmental damage^34^. Due to positive effects on plant yield^6^, indigenous PGPR have been developed as commercial inoculants, used to increase crop productivity^35^. Thus, it is interesting and necessary to continue the screening of rhizobacterial diversity for efficacious PGPR. In search of efficient PGPR strains, multiple traits related to PGP have been tested together during the initial screening process^10,36^. These tests are usually in vitro screenings for PGP traits and are always tedious and time-consuming. *Arabidopsis thaliana* has been developed as a model dicot plant for plant molecular biology^37^ as well as plant–microbe interactions studies^38–40^. Valuable information has been obtained about growth promoting interactions between *Arabidopsis* and PGPR isolated from a wide variety of plant species^41^. Thus, in this study, *A. thaliana* was used to screen for plant growth stimulating activity associated with rhizobacteria from a wide range of plant species. In our previous study, 446 ectophytic and endophytic rhizobacteria were isolated from various crop and uncultivated plant species^42^. The major goals of this work were to: (1) screen bacterial isolates *in-vitro*, through direct plant growth-promoting effects on *A. thaliana* at an early growth stage; (2) identify the most effective bacterial isolates by 16S rRNA gene sequence analysis; (3) investigate their effects on long term growth of *A. thaliana* under unstressed and salt stressed conditions and (4) investigate interactions between *A. thaliana* and selected strains through a GFP technique. Our results demonstrated that all four tested rhizobacterial strains were able to colonize *Arabidopsis* roots and promote root growth at various stages of plant development. To the best of our knowledge, this is the first description of endophytic rhizobacteria associated with bittersweet (*Solanum dulcamara*) and fall dandelion (*Scorzoneroides autumnalis*), able to promote plant growth in *A. thaliana*.

## Materials and methods

### Plant material, bacterial strains, and growth conditions

The wild type *Arabidopsis thaliana* Columbia (Col-0 accession) was obtained from the Arabidopsis Biological Resource Center (ABRC, Ohio State University, Columbus, OH, USA). Seeds were surface sterilized for 1 min in 70% ethanol and rinsed well with sterile ddH_2_O (3 times). The surface sterilized seeds were resuspended in sterilized ddH_2_O and stored at 4 °C in the dark for 48 h, to ensure stratification. Seed sterility was verified by incubating 20 seeds on Difco™ Nutrient broth (NB) agar at 28 °C for 3 days. For agar medium experiments, seeds were sown on 10-cm Petri-dishes containing one-half-strength (½) Murashige and Skoog (MS) (pH 5.7, adjusted with 10 mM KOH) supplemented with 0.8% (w/v) agar. All plates were covered and sealed with Parafilm and were arranged in a germination chamber maintained at 22 ± 2 °C with a 16 h light/8 h dark photoperiods, at a light intensity (photon flux density) of 100 μmole m^−2^ s^−1^. The plates were relocated to a new position in the germination chamber twice a week to control for position-dependent variation. Petri-dish experiments were organized following a completely randomized design and repeated three times, unless otherwise stated.

For plant-growth chamber experiments, *A. thaliana* seeds were either sown in individual Jiffy-7 peat pellets (Jiffy products, Plant Products Ltd., Brampton, ON, Canada) or at the soil surface at the rate of 4 seeds per pot in pots (100 mL) filled with nutrient-containing vermiculite-potting soil mixture that was autoclaved twice for 20 min at 121 °C, with a 24 h interval between autoclavings. Thinning of seedlings to 1 per pellet or pot was conducted after emergence of the first two true plant leaves (full germination, at 7 days). Plants were maintained in a growth chamber (Conviron PGR15, Winnipeg, MB, Canada) programmed for a 16 h photoperiod, day-night cycle with 200 μmol m^−2^ s^−1^ photosynthetic photon flux density, a constant temperature of 22 °C, and 60% relative humidity. Plants were irrigated twice a week with half-strength Hoagland nutrient solution and with ddH_2_O every 48 h. To avoid position effects in the soil-based experiment, plants were relocated, within the growth chamber, each week until sampling. All the experiments were conducted in a growth chamber, following a completely randomized design and repeated independently at least three times.

Isolated bacterial strains used for initial and secondary screening were routinely maintained on King’s B medium (KB). These ectophytic and endophytic bacteria were isolated from root samples of crops and uncultivated plants according to Qin et al.^43^, with modifications, from Sainte-Anne-de-Bellevue, which is located at latitude 45°24′22″ N, longitude 73°56′44″ W, in southwestern Québec, Canada. Single colonies were inoculated in liquid KB and grown aerobically on a rotary shaker (150 rpm) in the dark at 28 °C for up to 72 h, to reach the exponential phase of growth. Just before inoculation, bacterial cells were pelleted by centrifugation (6,000×*g*, 10 min, 4 °C), washed free of the growth medium 4 times with sterile 10 mM MgSO_4_, and then resuspended in 10 mM MgSO_4_ to a final density of 10^9^ CFU mL^−1^, as determined by optical density (OD) at 600 nm and serial dilutions with plate counts. These standardized suspensions were used for bacterization experiments. Control *A. thaliana* plants were mock-inoculated with sterile 10 mM MgSO_4_. Experiments related to phenotypic characterization of selected rhizobacteria were conducted three times, each with four replicates.

### Rapid bacterial screening assay

The effect of isolated bacteria on seedling growth in vitro was monitored as described by Subramanian^44^, with modifications. Approximately 100 newly isolated bacterial strains were used in this initial screening of potential plant growth promoting rhizobacteria. Sterilized *A. thalianas* seeds were incubated for 1 h at room temperature with a given bacterial cell suspension prepared as mentioned above, and with sterile 10 mM MgSO_4_ as the associated control^45^. Thereafter, 6 seeds were sown on ½ MS medium plates, comprising of 8 plates for each treatment. At 21 days after seeding (DAS), fresh weight (FW) of the whole rosettes was recorded.

### Secondary bacterial screening

Potential PGPR were selected through an initial screening and then subjected to a second-round of screening. In the in vitro assays on ½ MS medium, two application methods were employed. For seed treatment, surface sterilized *A. thaliana* seeds were incubated for 1 h at room temperature in 2 mL of bacterial suspension in 10 mM MgSO_4_, with sterile 10 mM MgSO_4_ as the control. For root tip inoculation^46^, 2 μL of bacterial cell suspension was pipetted onto root tips of 7-day-old seedlings, using equivalent amounts of sterile 10 mM MgSO_4_ as an axenic control. There were 10 replicate plates for each treatment and 6 seedlings in each plate. For total leaf surface area quantification, photographs were taken of each plate at 11, 15 and 20 DAS. Images were analyzed using the free Java image processing program ImageJ (https://rsb.info.nih.gov/ij; developed by Wayne Rasband, U. S. National Institute of Health, Bethesda, Maryland, USA). At 21 DAS, the control seedlings, and those inoculated with bacteria, were harvested and the whole rosette FW was determined. This experiment was conducted in quadruplicate.

### Petri plate assay for effects of bacterial inoculation on seedling growth

#### Germination activities of A. thaliana seeds after bacterization

Four isolates of rhizobacteria (referred to as n, L, K and Y) were selected from secondary screening on the basis of their stimulation effect on early seedling growth of *A. thaliana* plants*.* The finally chosen rhizobacterial isolates were used for all the following experiments. Studies have indicated that *Arabidopsis* seedlings cannot germinate or grow normally on MS medium with 200 mM NaCl or 400 mM mannitol^47,48^. Based on the results of our pre-experiments, 100 and 150 mM NaCl and 100 and 300-mM mannitol were chosen for evaluating the effects of the selected strains on *A. thaliana* plants growing under stressful conditions. Sterilized and stratified seeds were imbibed in the cell suspension of the chosen bacteria and were sown on ½ MS medium or medium supplemented with specific concentrations of NaCl and mannitol. Plates were transferred to a germination chamber with a light and dark cycle of 16 and 8 h at 100 μmole m^−2^ s^−1^ light intensity, at 22 ± 2 °C. Germination was defined as 1 mm protrusion of the radicle from the seed coat^49^. The number of germinated seeds was counted on a daily basis for a 10-day period. The percentage of germinated seeds was scored in four independent experiments, with 50 seeds per treatment.

#### In vitro osmotic stress

To test the effect of bacterial treatment on tolerance to osmotic stress, surface-sterilized *A. thaliana* Col-0 seeds were incubated in a bacterial suspension for 1 h in 10 mM MgSO_4_ or sterile 10 mM MgSO_4_ (for control) and were sown on ½ MS and allowed to grow for 3 days with the plates in a vertical orientation. Seedlings were then transferred, using sterile forceps, to ½ MS medium or ½ MS medium with added osmoticum (100 mM mannitol), and allowed to grow for 11 days. After this 11-day stress treatment, the seedlings were observed and photographed. Rosette areas were measured using ImageJ (NIH). There were 8 replicate plates for each treatment and 6 seedlings in each plate. The seedlings were collected and the whole plant and rosette FW were measured and recorded.

#### In vitro salt stress

To evaluate salt tolerance during early seedling growth^50^, surface-sterilized *A. thaliana* Col-0 seeds were incubated in a bacterial suspension for 1 h, after which seeds were imbibed on ½ MS agar plates comprised of control and 100 mM NaCl treatment, and allowed to grow for 20 days, after stratification at 4 °C in darkness for 3 days. There were 8 replicate plates for each treatment and 6 seedlings in the same plate. For total leaf surface area quantification, photographs were taken of each plate at 14 DAS. The seedlings were then collected and the whole plant (percentage growth reduction due to salinity, relative to control conditions) and rosette FW were recorded.

Chlorophyll content in leaves of control and rhizobacteria-treated *Arabidopsis* was quantified according to the method of Fan et al.^51^. Fresh leaf tissue (50 mg) was extracted in the dark with 1 mL of pure methanol to extract all the chlorophyll into solution. The homogenate was centrifuged at 10,000×*g* at 4 °C for 10 min and the corresponding supernatant was used for quantifying chlorophyll content. Absorbance was measured at 652, 665, and 750 nm using a Ultrospec 4,300 Pro UV/Visible spectrophotometer (Fisher Scientific, Canada). The amount of chlorophyll was calculated using the extinction coefficient, as indicated by Ritchie^52^. Concentrations of chlorophyll were expressed as μg mg^−1^ FW leaf tissue.

#### Root architecture analysis

Root system architecture was assessed on 14-day-old *A. thaliana* seedlings. In brief, surface-sterilized seeds were treated with chosen bacteria and sown on ½ MS medium supplemented or not with 100 mM NaCl and placed in a vertical orientation. The seedlings were grown for 2 weeks, and then images of root systems were recorded and analyzed using the ImageJ software (NIH) with the NeuronJ plug-in (https://www.imagescience.org/meijering/; developed by Erik Meijering, University Medical Center Rotterdam, NL). The primary and lateral root length for growth variables were measured.

### In planta bacterial impact on growth of *A. thaliana* in soil

#### In vivo assessment of plant-growth promotion

The ability of isolated rhizobacteria to promote plant growth was assessed in a controlled environment (growth chamber) with 18 plants per treatment using a completely randomized design. Briefly, surface-sterilized seeds were placed on the surface of sterilized potting soil in each pot, after which each seed was inoculated with 1 mL of a bacterial suspension in 10 mM MgSO_4_. For the control treatment, seeds were incubated with an equal amount of sterile 10 mM MgSO_4_. At 28 days after bacterial innoculation, *A. thaliana* plants were collected for measurement of shoot FW. Photographs were taken of each plant at harvest for total leaf surface area quantification. Fresh rosettes were dried in an oven at 85 °C for 48 h, and DW was recorded as mg g^−1^ FW. Rosette areas were measured using ImageJ (NIH). The effect of bacterial treatment on the growth of *A. thaliana* was also tested under non-sterile conditions (peat pellets). Furthermore, the long-term growth effects of bacteria on Col-0 were determined following a 49-day growth period. Rosette diameter and stalk length was measured at this time.

#### Salinity tolerance assay

Preliminary experiments showed that *A. thaliana* plants can tolerate 200 mM NaCl, while at 250 mM, stress symptoms were very severe (e.g. retarded growth, leaf senescence and loss of turgor) . Hence, thus 200 mM NaCl was chosen for this work. The ability of bacterial treatment to enhance plant resistance to salt stress was evaluated according to the methods of Zhang et al.^53^, with some modification. Briefly, developmentally uniform 6-day-old healthy seedlings were aseptically transferred from ½ MS agar plates, using tweezers, to pots filled with sterile potting soil, with one seedling per pot. After three days of acclimation, plants were root-treated with 10 mL of a given bacterial suspension, followed by root-flooding with 200 mM NaCl (at 25 mL plant^−1^), 10 days post bacterization. Then the plants were allowed to recover for a week by irrigating with sterile ddH_2_O, after which the plants were assessed for visual symptoms of salt stress. Relative chlorophyll content (or leaf greenness) was measured at the end of the experiment for all plants in each treatment, using a hand-held chlorophyll meter (SPAD-502, Konica Minolta). All plants were well irrigated before this measurement was taken. The fresh and dry weight of the aerial parts of plants were also measured.

#### Measurement of ROS scavenging activity

The antiradical activity of *A. thaliana* leaves (under normal growth conditions or salt stress) was determined using a DPPH (2,2-diphenyl-1-picrylhydrazyl hydrate) assay following Fan et al.^51^ with modifications. The antioxidants react with DPPH^•^ and convert it to 1,1-diphenyl-2-picryl hydrazine with decoloration (from deep violet to light yellow). The degree of decoloration indicates the scavenging potentials of the antioxidants in terms of hydrogen donating capabilities. Fresh leaf tissue (1.5 g) was homogenized in 15 mL pure methanol (MeOH) using a mortar and pestle. After centrifugation at 10,000×*g* for 10 min, the supernatant was recovered. The pellet was re-extracted with 10 mL MeOH. Supernatants were combined, and the total volume was made up to 25 mL. Each extract (150 μL) was added to 2,850 μL fresh DPPH methanolic solution (0.11 mM) and incubated in the dark for 1 h at 22 °C. Absorbance of the reaction mixture was read at 515 nm, against a MeOH blank. The scavenging activity was calculated using the equation: Inhibition % = [(Ab − As)/Ab]·100, where Ab is the absorption of the blank sample and As is the absorption of a treatment sample. The results were expressed as μM ascorbate equivalents (AE, μM ascorbic acid) g^−1^ FW through comparison against an ascorbate standard curve (0.01–0.8 mg mL^−1^).

For enzyme assays, approximately 0.2 g of leaves were ground into fine powder in the presence of liquid N_2_. The ground powder was then collected, using a spatula, into a microfuge tube with 1.5 mL of ice-cold 50 mM potassium phosphate buffer (pH 7.5) containing 1 mM ascorbate. The homogenate was vigorously vortexed for 10 min and then centrifuged at 10,000×*g* for 20 min at 4 °C. The supernatant (crude enzyme) was transferred to a new microtube, kept on ice, and analyzed right away. Protein concentration was determined by measuring the absorbance at 595 nm 5 min after mixing the solution with Bradford’s reagent. Bovine serum albumin (200–900 μg mL^−1^) was used as a reference standard.

Ascorbate peroxidase (APX, EC 1.11.1.11) activity was determined by following the decrease of ascorbate according to Nakano and Asada^54^ with modifications. The enzymatic reaction for APX was initiated by adding 50 μL of crude extract to the reaction mixture (1,372 μL of 50 mM potassium phosphate, 75 μL of 10 mM ascorbate, 3 μL of 100 mM H_2_O_2_). After 1 min at 25 °C, the H_2_O_2_-dependent oxidation of ascorbate to dehydroascorbate was monitored by measuring the decrease in absorbance at 290 nm. Nonenzymatic oxidation of ascorbate without enzyme extract was used as control. The amount of APX able to oxidize 1 μmol of ascorbate at 25 °C within 1 min is defined as 1 unit of enzyme.

Catalase (CAT, EC 1.11.1.6) activity was determined as a decrease in absorbance at 240 nm at 25 °C following the decomposition of H_2_O_2_. The assay mixture (1 mL) contained 50 mM potassium-phosphate buffer (pH 7.5), 20 mM H_2_O_2_ and 20 μL of enzyme extract. Phosphate buffer without H_2_O_2_ was used as the control. The activity (1 unit of CAT) was calculated as the amount of enzyme catalyzing the decomposition of 1 μmol of H_2_O_2_ for 1 min^55^.

For determination of peroxidase (POD, EC 1.11.1.7) activity, the reaction mixture (2 mL) consisted of 100 mM potassium-phosphate buffer (pH 6.0), 0.1 mM pyrogallol, 5 mM H_2_O_2_, and 10 μL of crude enzyme. After incubation at 25 °C for 5 min, the reaction was stopped by adding 1.0 mL of 2.5 N H_2_SO_4_. The absorbance (indigo color) formed by purpurogallin was read at 420 nm against a blank (Milli-Q water). One unit of POD forms 1.0 mg of purpurogallin from pyrogallol in 20 s at pH 6.0 and 20 °C.

#### Proline estimation

Proline content of fresh leaves from 4-week-old plants was determined following Singh and Jha^56^, with modificatioins. Fresh leaf tissues (100 mg) were homogenized in 3 mL of 3% sulfosalicyic acid at 95 °C for 15 min. After centrifugation at 8,000×*g* for 10 min, the resulting supernatant was combined with a 1:1:1 mixture of glacial acetic acid, 2% acid ninhydrin (1.25 g ninhydrin in 30 mL glacial acetic acid) and 20 mL 6 M orthophosphoric acid. The mixture was boiled for 30 min in a boiling water bath. After cooling at room temperature for 30 min, 1 mL of toluene was added to the mixture, with vigorous shaking for 30 s, to extract the red chromophore. The absorbance of the upper toluene phase was determined spectrophotometrically at 520 nm against toluene as a blank. Free proline content was measured by comparison with a pure L-proline standard curve prepared by the same method and calculated on a fresh weight basis expressed as mg 100 g^−1^ FW.

### Phylogenetic analysis of selected bacteria

The four selected isolated rhizobacteria were cultivated in KB medium at 28 °C for 12–72 h. The culture was used for DNA isolation by the DNeasy Blood & Tissue Kit (Qiagen) according to the manufacturer’s instruction. To determine the phylogenetic positions of the four strains, 16S rRNA gene sequneces for them were amplified using one set of universal 16S rRNA gene primers: 27F (5′-AGRGTTYGATYMTGGCTCAG-3′) and 1064R (5′-CGACRRCCATGCANCACCT-3′). The hypervariable regions V1 to V6 of 16S rRNA gene were covered. Genomic DNA was extracted with the DNeasy Blood & Tissue Kit (Qiagen) according to the manufacturer’s instruction. DNA sequencing of PCR products were carried out using the Genome Quebec Innovation Centre (McGill University) service on a 3730XL DNA analyzer systems (Applied Biosystems). Retrieved DNA sequences were edited by Seqman Pro (DNASTAR Lasergene package). DNA sequences were compared with other 16S rRNA gene sequences available within the BLASTN program (https://blast.ncbi.nlm.nih.gov/Blast.cgi) and with the sequences held in the EzTaxon-e server^57^, and then were aligned with similar sequences by using the CLUSTX program. All reference sequences were obtained from the National Centre for Biotechnology Information (NCBI) and Ribosomal Database Project (RDP) databases^58^. Phylogenetic trees based on 16S rRNA gene sequences were constructed by applying the neighbor-joining method using MEGA version 7.0 based on the Kimura 2-parameter model with 1,000 replicates of bootstrap values^59^.

The partial 16S rRNA gene nucleotide sequences of the four isolated bacterial strains n, L, K and Y have been deposited in the NCBI nucleotide sequence database and can be retrieved under the accession nos. from MG669245 to MG669248, respectively.

### GFP-tagging for in planta tracking

#### Preparation of competent cells

Electro-competent cells of strains n and Y were prepared according to the standard protocol for *Escherichia coli* with some modifications. Briefly, 10 mL of KB broth was inoculated with 100 μL of overnight culture and grown to an OD_600_ of 0.5. The cells were first chilled on ice for 15 min then centrifuged and washed repeatedly with ice-cold 10% sterile glycerol, and were then re-suspended in chilled water and pelleted and washed two times, after which the pellets were re-suspended in ice-cold 10% glycerol, divided into 40 μL aliquots, and stored at –80 °C.

For strain L, chemo-competent cells were prepared following the method of Anagnostopoulos and Spizizen^60^, with some modifications. In brief, overnight culture of strain L in LB medium was diluted 50-fold in growth medium (per 100 mL ddH_2_O: 0.54 g KH_2_PO_4_, 1.26 g K_2_HPO_4_*·*H_2_O, 0.09 g trisodium citrate*·*2H_2_O, 2 g glucose, 0.2 g potassium glutamate, 1.1 mg ferric-ammonium-citrate, 5 mg l-tryptophan, 36 mg MgSO_4_) and grown at 37 °C (200 rpm) until OD_600_ reached 1.0, from which 400 μL aliquots were made and frozen at –80 °C.

#### GFP plasmid transformation

Broad scale GFP tagging was attempted with a wide-host promoter plasmid, pDSK-GFPuv (obtained from Dr. Kiran Mysore at The S. R. Noble Foundation). This binary plasmid was used to transform chemically competent cells of *E. coli* DH5α (Invitrogen), which were then stored at –80 °C. Strains n and Y were electroporated with the plasmid extracted from *E. coli* via standard protocol, as described by Wang et al.^61^. Briefly, 40 μL of cold competent cells were mixed with 4 μL of plasmid DNA (250 ng μL^−1^) and then electroporated using an Eppendorf Multiporator®.

For strain L, competent cells were transformed by mixing with 600 ng of plasmid DNA in a transformation mixture (per 100 mL ddH_2_O: 2.5 g yeast extract, 4 g casamino-acids, 0.025 g l-tryptophan) and incubating at 37 °C, 150 rpm for 1 h. Transformants were selected on kanamycin (35 μg mL^−1^) KB agar plates. The expression of GFP was analyzed by visualization under fluorescence microscopy.

#### Microscope analysis of root colonization

The overnight cultures of GFP-tagged strains were diluted and grown in KB liquid medium at 28 °C, to the exponential growth stage. Cells were collected by centrifuging at 5,000×*g* and pellets washed twice in 10 mM MgSO_4_. The number of viable cells (10^9^ CFU mL^−1^) for each strain was determined by plate counting on KB agar plates containing 50 mg L^−1^ of kanamycin. To verify root-endophyte habit, that is, the ability to endophytically colonize in *Arabidopsis*, seeds of *A. thaliana* were surface sterilized and treated with GFP-labelled bacteria as described above. Seeds treated with 10 mM MgSO_4_ were used as a negative control. After growing vertically in ½ MS agar medium for 14 days, plantlets were washed with ethanol and sterile water to remove unbound bacteria, after which the entirety of the roots were examined. Light and fluorescence microscopy were performed with a Leica fluorescent microscope and Northern Eclipse software. Observations of unlabeled seedlings provided information on the auto-fluorescence level of the sample. The experiment was repeated twice and representative images of at least 20 roots of each treatment (inoculated or non-inoculated) was collected. Final pictures were generated through ImageJ (NIH) by merging the two channels.

#### Bacterial re-isolation in Arabidopsis

Five individual *A. thaliana* Col-0 plants treated with GFP-marked bacterial isolates (strains n, L and Y) or original bacterium (strain K) were harvested for collecting the shoot (strains n, L and Y) or root samples (strain K) 14 days post inoculation. Control seedlings were seed-treated with 10 mM MgSO_4_. Endophytic bacteria were isolated following the procedure described by Qin et al.^43^. Briefly, after appropriate sterilization to remove possible epiphytes, rosette or root samples were mixed and ground in sterile 0.8% NaCl and vortexed for 10 min. Serial dilutions of the supernatant from rosette or root tissues were plated on KB medium supplemented with appropriate antibiotics. After incubation for 24 to 72 h at 28 °C, inoculated agar plates were observed. Colony-Forming Units (CFUs) on these plates were counted thereafter. Identity of each isolate was confirmed by using the EzTaxon-e server^57^ based on 16S rRNA gene sequencing.

### Statistical analysis

For all experiments, the overall data were analyzed by one-way analysis of variance (ANOVA) and differences between control and bacteria-treatments were considered statistically significant at the *P* ≤ 0.05 level using Tukeys Honestly Significant Differences (HSD) test of the COSTAT® statistical software (CoHort Software, Monterey, CA, USA). Standard errors were calculated for all mean values.

## Results

### Bacterial screening for growth promotion of *Arabidopsis*

**Figure 1:**
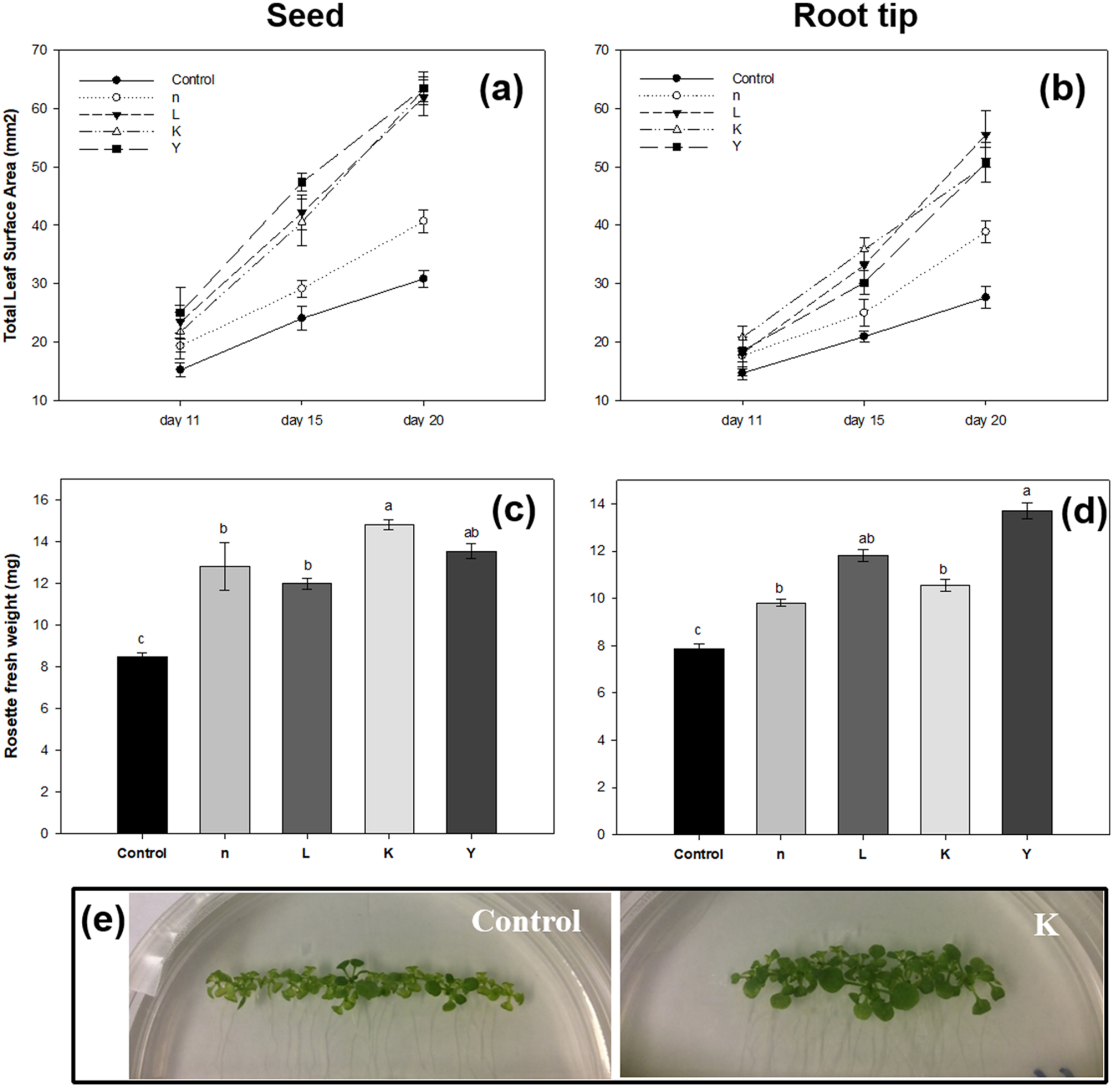
Effects of seed or root tip treatment of *A. thaliana* wild-type Col-0 with rhizobacteria on early seedling growth. The total leaf surface area (**a**,**b**) and rosette fresh weight (**c**,**d**) of *A. thaliana* plants inoculated with the 4 selected rhizobacteria strains after a 3-week growing period on agar plates. Pictures (**e**) are phenotype of Col-0 plants treated with strain K after a 3-week growing period on vertical agar plates. Different letters indicate statistically significant differences between treatments (*P* < 0.05). Bars indicate mean ± standard error from three independent experiments, with 20 seedlings per treatment.

In order to find potential PGPR from newly isolated rhizobacteria, we conducted an in vitro study to screen for the plant growth promoting effects of isolated strains by measuring various physiological parameters of *A. thaliana* Col-0. A total of 98 isolates were selected based on their origins and taxonomical diversity for the initial screening on *Arabidopsis*. While some isolates (e.g. B58, K2, Y9 and L1) showed no significant stimulation effect on plant growth, others promoted shoot growth (Fig. S1). Based on the above-ground fresh weight (FW) of *A. thaliana* seedlings after 21-day of growth, 12 potential isolates (Fig. S1) which caused significant enhancement of shoot growth (from 1.3 to 2.5-fold increase over the control), were chosen for a second screening test. Only those that clearly showed plant-growth-promoting effects in two independent experiments were selected for further research. Four out of 12 selected isolates showed consistent and significant growth promoting effects compared to controls and other isolates. In vitro bioassays showed that treatment of seeds or root tips of *A. thaliana* with rhizobacteria n, L, K and Y significantly increased seedling growth, relative to controls, after 21 days of seedling growth (Fig. 1). For example, the rosette fresh weight (root-tip treated) was significantly increased (by approximately 20, 43, 30, and 60%, respectively) by strains n, L, K and Y, respectively, as compared to control plants that were not exposed to the bacteria. Inoculation of root-tip by strains L, K and Y, respectively, led to a ca.77% increase in the total leaf surface area versus the mock-inoculated control.

### Molecular taxonomy of candidate bacterial strains by 16S rRNA gene sequencing

To determine the relatedness of strains at the genetic level, the selected bacterial isolates n, L, K and Y were characterized by partial 16S rRNA gene sequencing. Results were from the comparison of the nucleotide sequences with deposited sequences in GenBank using the Mega BLAST algorithm. Similarities of the 16S rRNA gene sequences revealed that strain n showed high identity with *Pseudomonas* and clustered with *P. koreensis* Ps 9-14^T^, based on 99.8% similarity.Strain K was from the phylum *Bacterioidetes* and from the family *Sphingobacteriaceae* and grouped with *Mucilaginibacter lappiensis* ATCC BAA-1855^ T^, with identity value > 97%. Strain L clustered with *Bacillus mobilis* 0711P9-1^ T^ from the family *Bacillaceae* and strain Y with *Rhizobium jaguaris* CCGE525^T^ from the Rhizobiaceae family. The latter two strains had 100% identity with the 16S rRNA gene sequences of their corresponding type strain (Table 1). The identification of selected endophytic rhizobacteria encoded n, L, K and Y most closely resembled *Pseudomonas* sp., *Bacillus* sp., *Mucilaginibacter* sp. (97.2% confidence) and *Rhizobium* sp., respectively (Table 1).

**Table 1:** Molecular identification of rhizobacterial isolates using 16S rRNA gene as query sequences.

Strain code	Closet relative (NCBI)	Closest type strain (accession number)	% Similarity (EzTaxon)	Origin
n	*Pseudomonas* sp.	*Pseudomonas koreensis* (AF468452)	99.76	*Phalaris arundinacea*
L	*Bacillus* sp.	*Bacillus mobilis* (KJ812449)	100.00	*Solanum dulcamara*
K	*Sphingobacteriaceae* bacterium kmd_018	*Mucilaginibacter lappiensis* (jgi.1095764)	97.21	*Scorzoneroides autumnalis*
Y	*Rhizobium* sp.	*Rhizobium jaguaris* (JX855169)	100.00	*Glycine max*

A neighbour-joining phylogenetic tree of the isolated strains n and K was constructed by combining the sequences of the specific strain with its closest relatives (Fig. S2). Phylogenetic tree data suggested their relatedness with several other strains of *Pseudomonas* and *Mucilaginibacter* sp., respectively. All the four isolates showed 97% to 100% similarities with type strains of two or more species within the corresponding genera, thus the species affiliation for all the 4 isolated strains was not defined.

### In vitro response of treated *Arabidopsis* to salt and osmotic stress

Firstly, to check whether bacterial treatments affected the germination activities of *A. thaliana*, the seeds were germinated on the ½ MS medium containing varying concentrations of NaCl or mannitol and the germination rates were compared. There were no significant differences among the control and treated plants when germinated under optimal conditions (Fig. S3). In the presence of exogenous NaCl or mannitol, the germination of both control and bacterium-treated seeds was inhibited significantly. For example, at the 4th day after sowing on ½ strength MS agar medium supplemented with 150 mM NaCl, only approximately 10% of the control and treated seeds germinated (Fig. S3). We also observed that the germination of treated seeds was comparable to the controls under stress conditions (Fig. S3). These results indicated that rhizobacterial treatment of *Arabidopsis* did not affect plants at seed germination stage.

**Figure 2:**
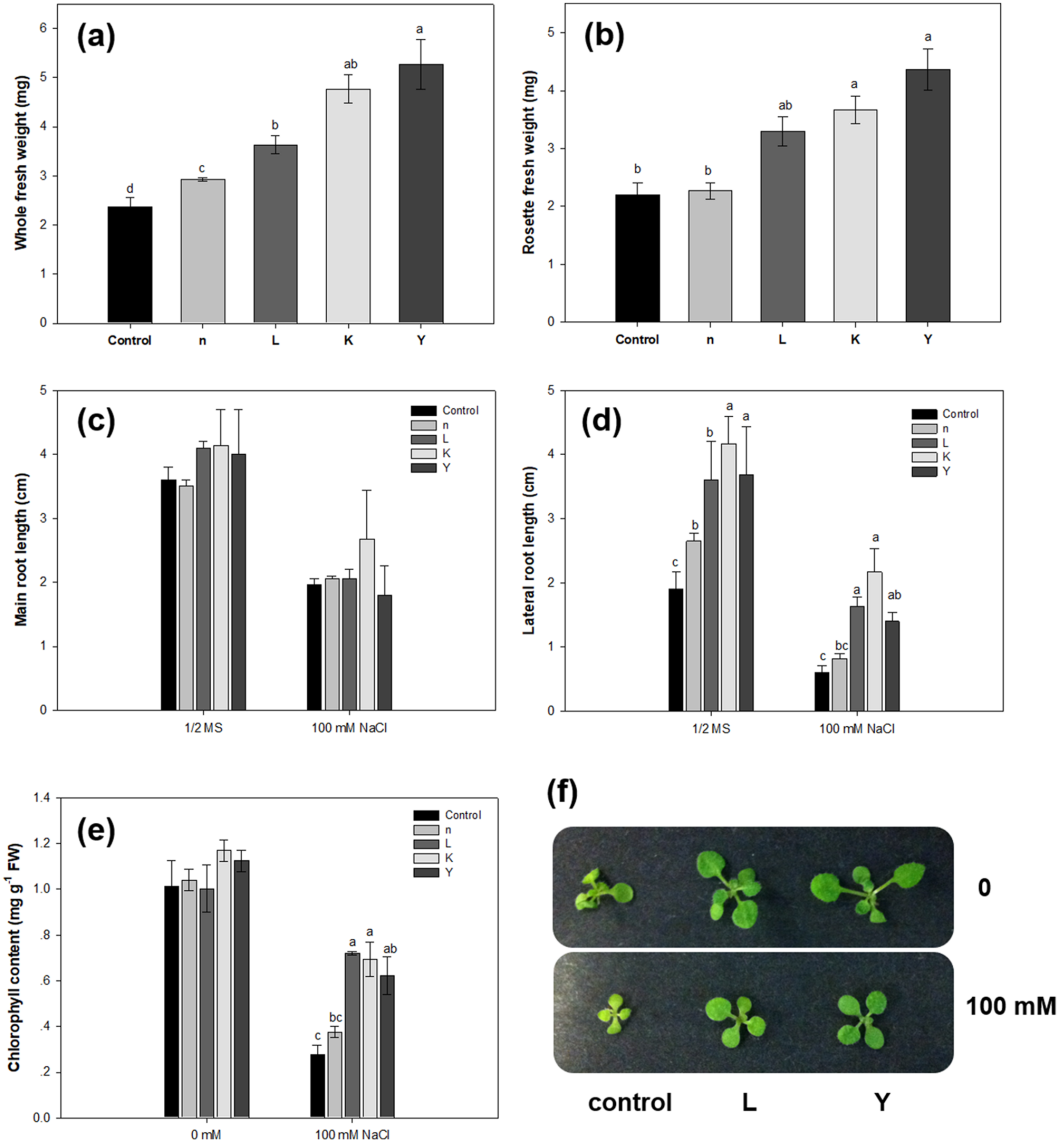
The growth of *A. thaliana* plants under salt stress conditions. Surface-sterilized seeds were treated with bacteria suspension for 1 h and sown onto ½ MS agar medium supplemented with 100 mM NaCl. Total fresh weight (**a**), rosette fresh weight (**b**), and root length (**c**,**d**) of *A. thaliana* seedlings were measured after 14 days of co-cultivation with the indicated bacterial treatment. (**e**) Assay of chlorophyll content in leaves of bacteria-treated seedlings. (**f**) Phenotype of Col-0 plants treated with the rhizobacteria after a 3-week growing period vertically. For root length measurement, the average value for 20 plantlets per treatment was determined for each replicate. Vertical bars represent the mean and error bars represent standard error based on three independent experiments. Different letters indicate statistically significant differences (*P* < 0.05).

Subsequently, growth variables such as root length, chlorophyll content, and fresh weight were measured and analyzed to observe the effects of biopriming on the early seedling growth of *A. thaliana* plants under salt stress. When *A. thaliana* seeds were primed with bacterial strains n, L, K and Y, the seedlings grew better than controls during the first 14-day of growth, which is consistant with our earlier observation that the total rosette fresh weight was increased by 20–50% following inoculation with the selected four strains. The growth of both treated and control seedlings was significantly repressed after a 14-day growth on ½ MS media containing 100 mM NaCl, but the growth of the treated plants was much less inhibited, compared to the controls (Fig. 2). Inoculation with all four strains led to 23.6 to 122.36% increases in whole plant biomass (Fig. 2a). The average lateral root lengths of the seedlings treated with strains n, L, K, and Y were significantly increased under normal conditions, being 1.36-, 2.71-, 3.61-, and 2.33-fold longer than the control, respectively (Fig. 2d); while under 100 mM NaCl treatment, the lateral root length of strains L, K and Y was significantly longer than the NaCl control. Bacterial inoculation resulted in no significant change in the primary root length under either normal or salinity stress conditions (Fig. 2c). Rosette fresh weight was maximum where the inoculation of strains L, K and Y was employed (Fig. 2b). Likewise, we observed that NaCl caused obvious decreases in total chlorophyll in control plants as compared to that of treated seedlings (Fig. 2e), but total chlorophyll was increased significantly in plants inoculated with strains L, K and Y compared to non-inoculated salinized plants. Overall, these results suggested that, in our culture conditions, bacterial treatments enhanced tolerance to salt stress and stimulated lateral root growth rate during early seedling growth of *Arabidopsis* on agar plates.

**Figure 3:**
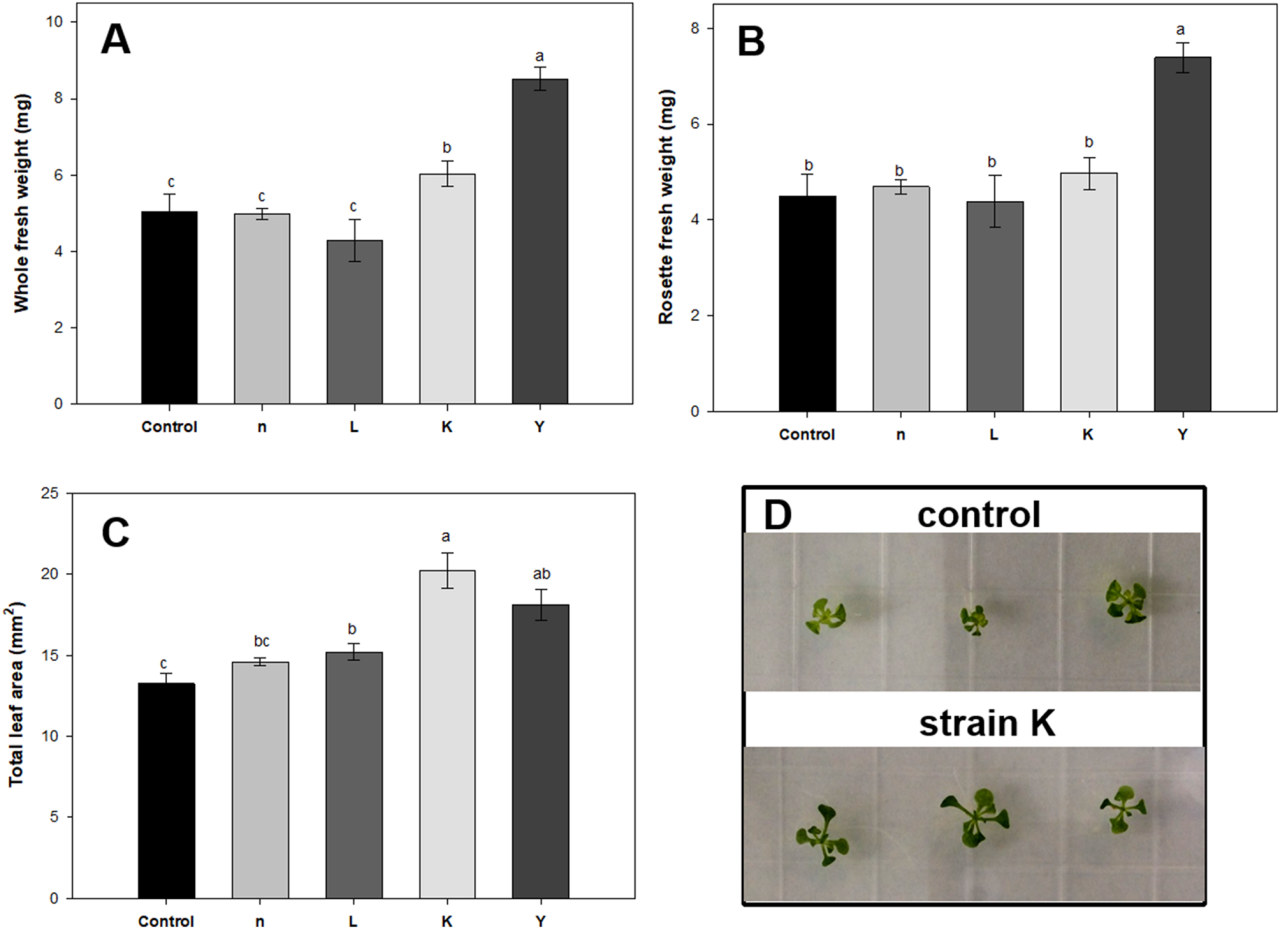
The growth of *A. thaliana* plants under osmotic stress. Surface-sterilized seeds were treated with bacteria and sown on ½ MS agar medium and allowed to grow for 3 days. Seedlings were then transferred to ½ MS medium or ½ MS medium with 100 mM mannitol added and allowed to grow for 11 days. Bacterial treatment triggered enhancement of plant growth as characterized by (**A**,**B**) total fresh weight and rosette fresh weight and (**C**) total leaf surface area per plant. Representative images of plants (**D**) with bacterial treatment or 10 mM MgSO_4_ as control. The results are mean ± standard error from three independent experiments. Different letters indicate statistically significant differences (*P* < 0.05).

As it is known that NaCl imposes both ion toxicity and osmotic stress in plants, here we used mannitol in ½ MS medium to check for the osmotic stress effect on seedling growth in *A. thaliana*. At the early stage of observation, after 14 day of growth, we found that the growth of control seedlings was more hampered than bioprimed ones (Fig. 3). The average leaf area of seedlings treated with strains L, K and Y were 1.3, 1.8 and 2.2-fold higher than the control under 100 mM mannitol, respectively (Fig. 3c). Another measurement showed that seed treatment with strains K and Y not only resulted in higher leaf surface areas but also caused higher seedling fresh weight, as compared to controls (Fig. 3), indicating that plants treated with strains K and Y showed better adaptation to the dehydration stress.

### Bacteria confer facilitated growth and increased salt resistance in planta

**Figure 4:**
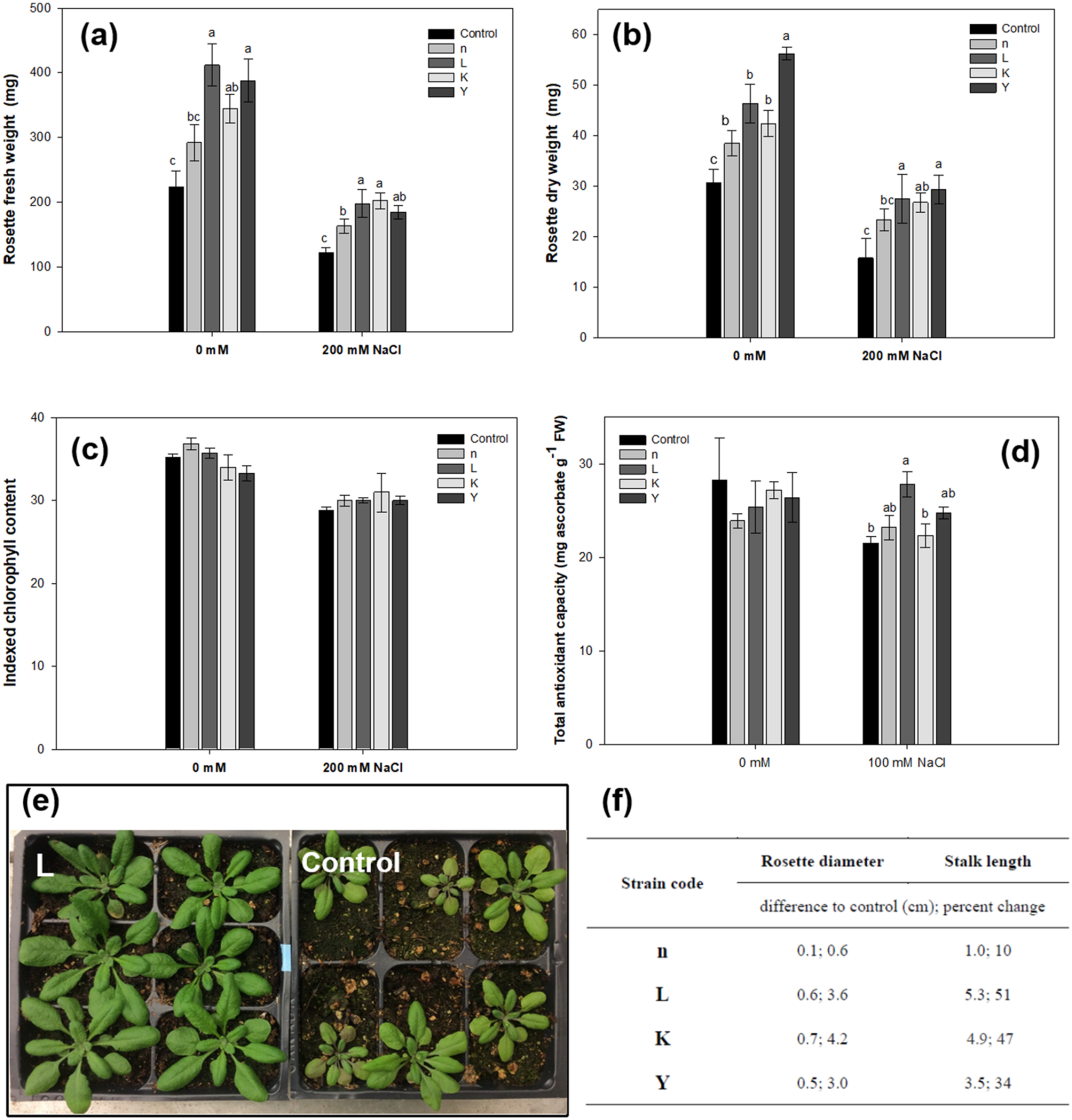
The effect of bacteria on growth and salt tolerance of *A. thaliana*. The average rosette fresh weight (**a**), dry weight (**b**), chlorophyll content (**c**) of *A. thaliana* plants root-inoculated with the rhizobacteria strains after a 4-week growing period in soil (0, 200 mM NaCl). (**d**) Reduction of DPPH by *A. thaliana* seedling extracts. Protein extracted from the leaves of 4-week-old plants were used in DPPH scavenging activity. (**e**) Representative plants grown in sterile soil with salt stress. *A. thaliana* specimen were photographed after one month. The results are mean ± standard error from three independent experiments. Different letters indicate statistically significant differences (*P* < 0.05). 6-day-old *A. thaliana* seedlings germinated on ½ MS plants were transferred into pots and then treated with bacterial cell suspension or 10 mM MgSO_4_. After 3 days, seedlings were irrigated with either 0 mM or 200 mM NaCl. After three weeks in pots, the *A. thaliana* specimens were photographed. The aerial parts were then taken for fresh weight and dry weight (85 °C for 2 days) measurement. (**f**) Long-term growth effects of rhizobacteria on *A. thaliana* (non-sterile peat pellet). Plants were grown in a growth chamber with 12 h day (22 °C) and 12 h night (20 °C). Measurements of rosette diameter and stalk length were taken after 49 days growth.

The above results showed that pre-incubation with selected rhizobacteria significantly increased early seedling growth on MS agar plates, with full nutrition, under sterile conditions. However, the in vitro assay is not a good measure of long-term growth determinations as the seedlings are within an enclosed environment, which could induce other stresses over the long term. Thus, the effect of bacteria on plant long-term growth in pots was tested. In brief, 10-day-old seedlings of *A. thaliana* were inoculated with one of the four selected rhizobacteria and were then grown for a 28-day period in a growth chamber. The four tested strains presented different PGP abilities and nearly all of them had considerable impact on different growth variables of *A. thaliana*, compared with the negative control. Growth promotion was first assayed using sterilized potting mixture. Under stress-free conditions, the lowest amount of rosette fresh weight was found in the control treatment, while the total leaf surface area was clearly increased (by ca. 57%) by bacteria L, K and Y, as compared to control plants. Moreover, the rosette fresh and dry weights were also significantly increased by the same treatments (Table S1). These results were in line with those from the in vitro experiments above-mentioned. Secondly, plant growth was assayed on non-sterile peat substrates. Again, the bacteria significantly enhanced growth of 28-day-old plants in terms of rosette fresh and dry weight as well as total leaf area (Table S2). As for long-term effects, bacteria significantly enhanced growth of 9-week-old plants. Stalk length of *A*. *thaliana* plants exposed to bacterial strains L, K and Y was significantly increased, by 51% after 49 days, as were rosette diameters, by 3.4% (Fig. 4f). Taken together, these results demonstrate that bacteria efficiently boosted the vegetative growth of *A*. *thaliana* on various substrates under both sterile and non-sterile conditions (stress-free).

To further test whether bacteria modulated plant responses against abiotic stress in vivo, we infected *A*. *thaliana* seedlings and monitored the growth behavior after a salt shock with 200 mM NaCl by capillary process from the bottom of the pots. Then, the plants were allowed to recover for 7 days by regular irrigation with sterile ddH_2_O. Under these moderate salt stress conditions, all plants survived. After one-month of growth in pots, the most prominent effect of inoculation in the presence of NaCl was observed in terms of fresh and oven dry weight of rosettes (Fig. 4a,b). Fresh weight was increased significantly in response to inoculation with all four strains (1.5 – 2-fold higher than the uninoculated control). A similar effect was observed in the case of rosette oven dry weight, for which inoculation of strains L, K and Y resulted in rosette dry weight ca. 1.67-fold greater than other treatments (Fig. 4a,b). The combined results from both stress-free and salt stress conditions strongly suggested that the selected bacteria, especially strains L, K and Y, promoted growth and alleviated salinity stress in *A*. *thaliana*. The total chlorophyll contents were also measured on the 28th DAS. The results showed that the chlorophyll content in the rosettes decreased due to salt stress, but its content in both control and treated plants under normal or salt stress conditions were not significantly different (Fig. 4c).

**Figure 5:**
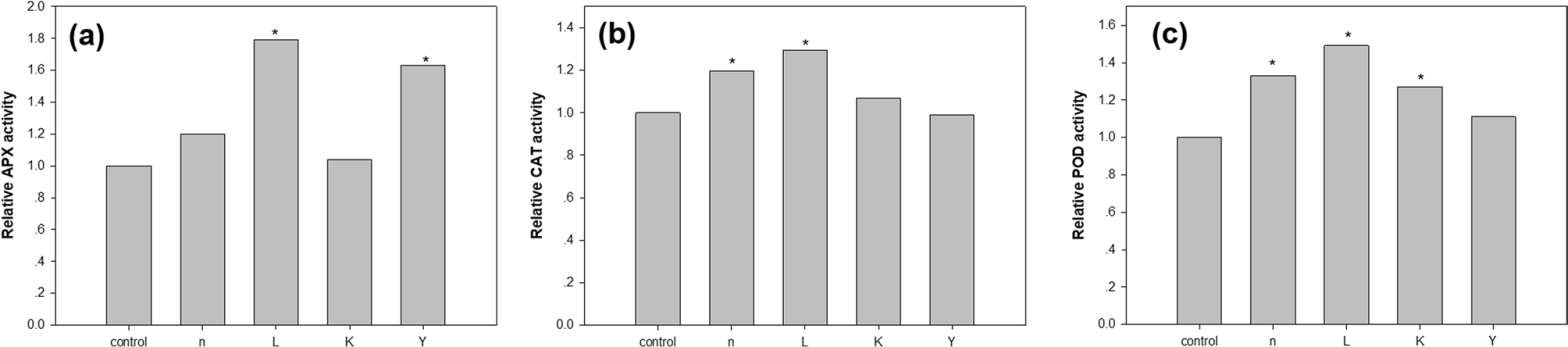
Effect of bacteria inoculation on the activities of (**a**) APX, (**b**) CAT and (**c**) POD in *A. thaliana* supplemented with 200 mM NaCl. Results are expressed as enzyme activity relative to untreated controls, are given as mean from three independent experiments. Asterisks (*) indicates statistically significant differences from control plants (*P* < 0.05).

Since salt stress exposure can cause oxidative stress and produce reactive oxygen species (ROS), i.e., superoxide, siglet oxygen, and hydrogen peroxide, the beneficial effects of bacteria on stress tolerance of *A*. *thaliana* was further explored. To study the metabolic mechanism behind the enhancement of salt-stress tolerance in bacteria treated plants, the accumulation of certain osmoprotectant molecules and ROS scavenging systems in plants was measured under both normal and salt stress conditions. We first measured the total antioxidant capacity (the DPPH assay). The ability of the leaf extracts of *A*. *thaliana*, treated with bacteria, or not, to act as donors of hydrogen to transform the DPPH radical (deep purple) into DPPH-H (decolorization) was investigated. The total antioxidant capacity of *A. thaliana* is given in Fig. 4d, showing that DPPH was neutralized more in plants treated by strain L (ca. 50%) than in untreated controls, under salt stress. Interestingly, treatments by strains n, K and Y (with a non-significant trend to increase) did not exert significantly increased total antioxidant capacity under either normal or stress conditions. Secondly, the activities of antioxidant enzymes were measured, showing significant enhancement in the activities of APX, CAT, and POD in inoculated plants, compared to respective control plants. The level of APX increased by 79% in salt-stressed plants inoculated with the bacterial strain L, and 63% by strain Y (Fig. 5A). In addition to APX, the level of the other two antioxidant enzymes also showed significant increases in plants treated with selected strains under salt stress. Specifically, in the presence of bacterial inoculation, CAT activity was significantly increased by 19.8 (strain n) and 29.6% (strain L) at 200 mM NaCl stress, compared to the controls (Fig. 5b). Bacterial inoculation with strains n, L and K led to increases (33, 49 and 27%, respectively) in POD activity, as compared to uninoculated plants under salt stress (Fig. 5c). There were no significant differences in the activities of all three enzymes, under normal conditions, among treatments and the respective control (Table S3).

**Figure 6:**
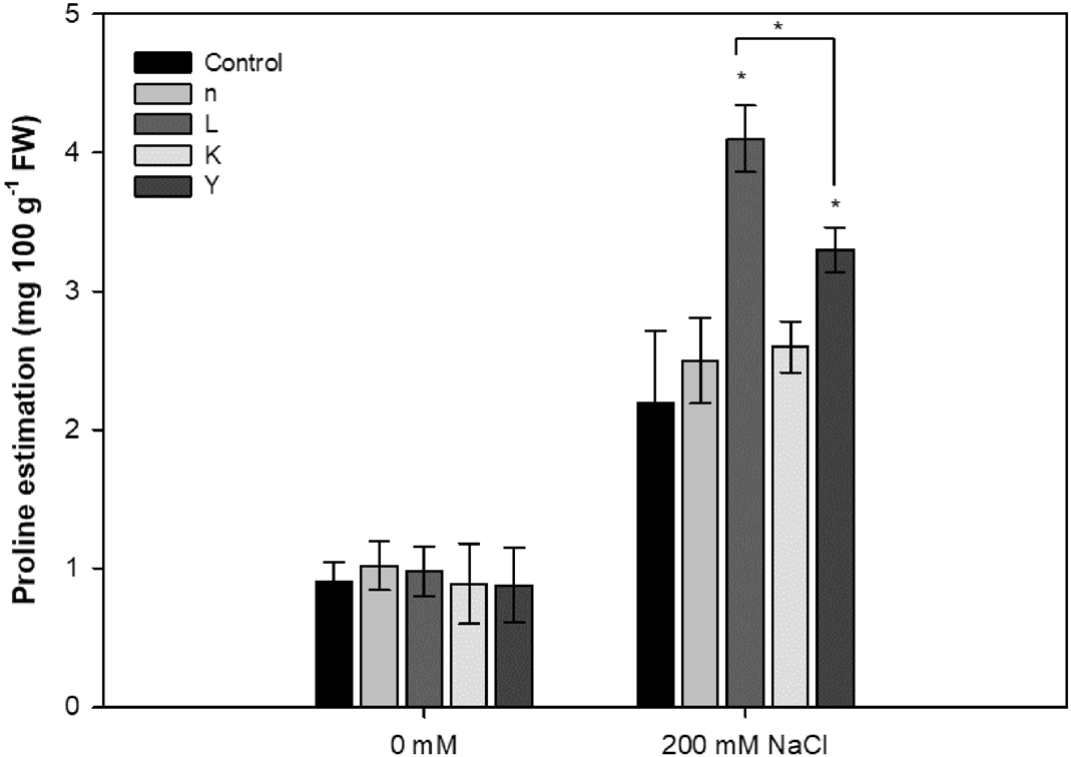
Effect of bacteria inoculation on the production of proline in *A. thaliana* supplemented with 200 mM NaCl. Values are given as mean ± standard error from three independent experiments (* *P* < 0.05).

Osmotolerance is induced by accumulation of compatible solutes to protect cells against salt-induced cell injury. Thus, the content of the osmoprotectant molecule proline within leaf tissues was measured, showing comparable results in both inoculated and control plants but significant augmentation after salt stress in plants treated with strains L (89%) and Y (46%) (Fig. 6).

### Spatial distribution of rhizobacteria in *Arabidopsis*

**Figure 7:**
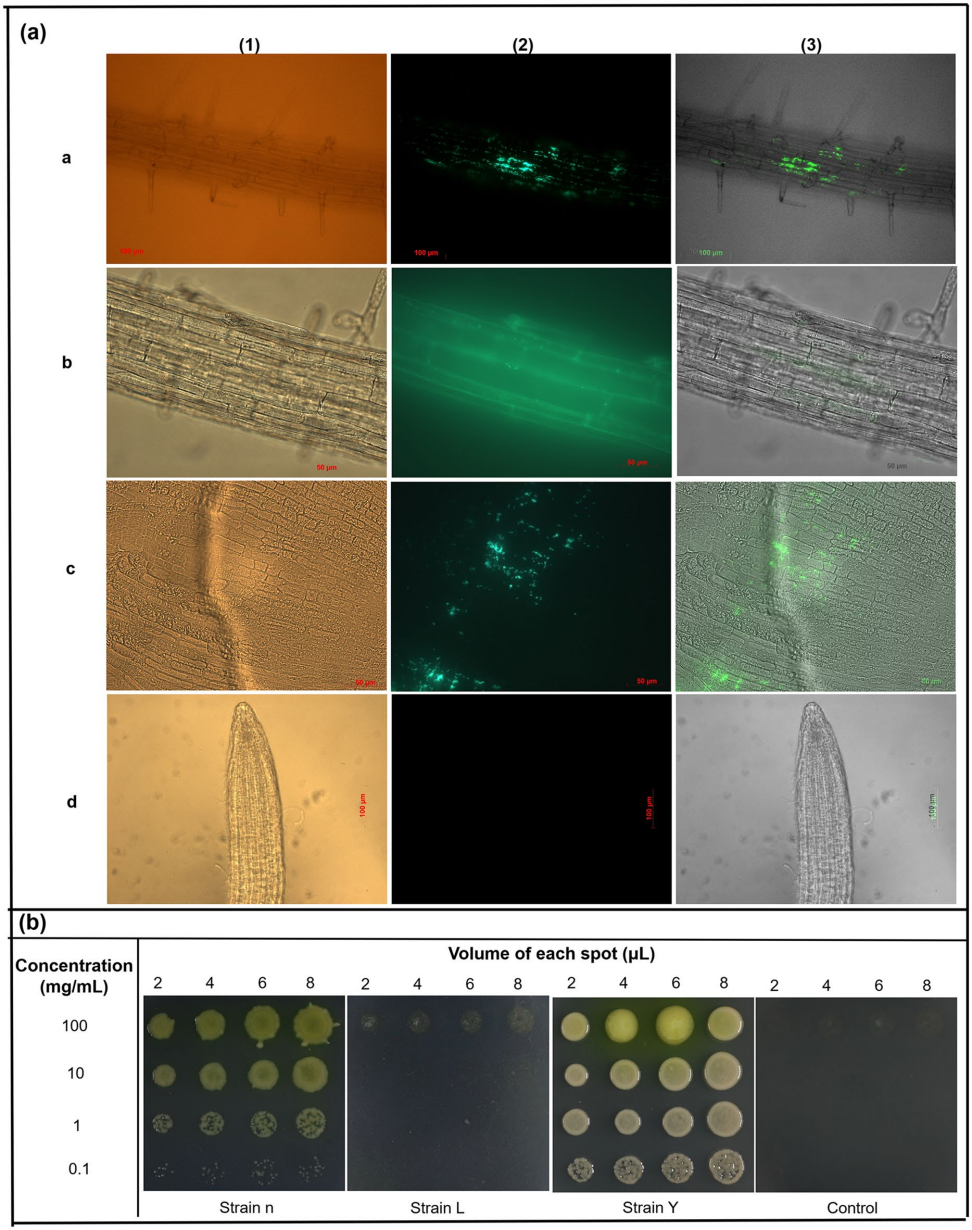
The persistence of recombinant rhizobacteria in roots and leaves of *Arabidopsis* wild-type Col-0. The transformed bacteria were inoculated onto *A. thaliana* seeds before plating on ½ MS agar medium. (**a**) Fluorescent microscope of a root was colonized endophytically by GFP-tagged bacteria. Roots were surfaced-sterilized with ethanol and sterile water before being observed. Cells of (a) *Pseudomonas* sp. (strain n), (b) *Bacillus* sp. (strain L), and (c) *Rhizobium* sp. (strain Y) were observed inside of roots 14 days after primed with selected strains: focus at maturation zone of the root as seen by the focused xylem strand. (d) control roots, from uninoculated seedlings. For each image set, the first panel (1) refers to phase contrast microscopy of *A. thaliana* root; the second panel (2) shows the corresponding image acquired under fluorescence light; the third (3) results from the merge of both images (1st and 2nd). (**b**) Leaf tissue was colonized endophytically by GFP-tagged bacteria (n, L and Y). Serial-diluted leaf supernatant were spot-inoculated onto KB agar medium supplemented with kanamycin. Observation was taken after incubation for 12 to 36 h at 28 °C.

Fusing reporter genes, such as the green fluorescent protein (GFP), to plasmids which are used to transform selected bacteria, has considerably advanced our knowledge of the localization of rhizobacteria within plants. In vivo microscopy gives more precise insights into the cellular localization of rhizobacteria. Thus, in this study, the pattern of root and shoot colonization (Col-0) was investigated by using recombinant bacterial strains expressing GFP and fluorescent microscopy, in order to evaluate whether the selected bacterial strains originally isolated from the interiors of roots of different crop and wild plants could colonize the internal tissues of *A*. *thaliana*. Attempts were made to GFP tag all four isolates; but, only three isolates could be successfully transformed with the GFP-harbouring plasmid (pDSK-GFPuv) (Fig. S4). Though GFPuv produces 45-fold brighter green fluorescence in *E. coli* than other GFP variants, and pDSK-GFPuv is shown to express *GFPuv* at high levels^61^, we could clearly see that transformed strain L (*Bacillus* sp.) showed lower green fluorescence than strains n and Y; the underlying reason is not known. When *A*. *thaliana* was treated with wild-type strains n, L, and Y, no fluorescent signal was detected throughout the entire root system. Two weeks after seed treatment with bacterial suspension of GFP-expressing strains n, L and Y, seedlings exhibited GFP signals in various tissues (i.e. apoplastic regions and the cytosolic compartment) of the maturation zone of the roots (Fig. 7a), demonstrating that these species are root associated. Extensive screenings of roots from 20 seedlings inoculated with GFP-carrying bacteria n and Y revealed abundant individual bacteria and/or bacterial colonies inside the roots (Fig. 7a), suggesting that these strains can colonize inside the roots of *A. thaliana*. It should be noted that, only a small number of GFP-labeled cells was observed inside the roots of *A. thaliana* treated with GFP-labelled strain L, indicating that strain L only sparsely colonizes inside the roots.

Epiphytic growth of all four strains along root tissues after one week of co-cultivation was visualized on agar medium. Re-isolation experiments indicated that there was transport throughout the entire plantlets and bacterial occurrence of strains n, L and Y appeared to be in both roots and shoots (Fig. 7b). Plate culturing confirmed that these four strains were present in both roots and leaves; with the endosphere of *A. thaliana* roots harboring approximately 10^3^ more bacteria than the rosette endosphere (Table S4). This again confirmed the endosymbiotic origin of the four selected rhizobacteria from their host plants and demonstrated their ability to colonize the interior parts of both roots and shoots of *A. thaliana* seedlings and then exhibit endospheric properties.

## Discussion

Plant microbiome communities (the phytomicrobiome) consist of large numbers of bacteria (ectophytes and endophytes) providing critical and sustainability benefits of improved soil health and nutrient utilization and increased plant growth and development. Although numerous bacterial strains are already at least reasonably well-understood in this capacity^6^, until our previous study^42^ the rhizobacterial diversity of different crop plants as well as wild plants in unmanaged fields in Québec remained largely unknown. In the present study, a total of 98 randomly selected rhizobacterial strains were first screened for short-term PGP effects on *Arabidopsis* seedlings in vitro and four of them were found to show excellent growth stimulating effects in initial screening; these four promising strains were then selected for further studies.

This is very intriguing given the small amount of sampling. We initially screened rhizobacteria under unstressed conditions, rather than abiotic or biotic conditions, based on two thoughts. Firstly, screening under stress conditions has often been linked to the natural biotic and abiotic status of host plants. For example, potential PGPR, isolated from plants grown under salinity stress conditions, were screened under salt stress *in planta*^62^. Secondly, far more PGPR might be able to promote plant growth under stress conditions than un-stressed conditions, as has been previously observed, which will make strains initially screened under optimal conditions of particular interest. Thus, we hypothesized that there might be some rhizobacteria which can exert plant growth promotion even under more optimal growth conditions, and this turned out to be true, as was also evidenced by Huang et al.^63^ and Takashita et al.^64^. Seed treatment, as well as root inoculation, with the four selected bacterial isolates, led to consistent positive performance in Petri dish assays (sterile).

Thus, soil experiments (*in planta*) in the growth chamber, which included growth to various developmental stages and under a range of growth conditions (initially sterile or non-sterile) and added robustness to our understanding of the PGP effects of these four isolates, were performed. Salinity is a very common abiotic plant stressor, severely affecting plant growth and crop production worldwide^65^. Thus, in the present study a Petri dish assay was performed using *A. thaliana* seeds inoculated with the selected strains and showing significantly enhanced plant growth under salt stress (100 mM NaCl) with regard to rosette fresh weight, seedling root length and total chlorophyll. Their ability to increase plant growth and ameliorate salt stress was further tested in a pot experiment. Inoculation with the isolated strains, especially L and K, significantly increased shoot and root growth and total antioxidant capacity in leaves of *Arabidopsis* under 200 mM NaCl. Leaf chlorophyll concentration is an indicator of salt tolerance. Chlorophyll is destroyed due to ions (Na and Cl) or reactive oxygen species (ROS), resulting in the degeneration of cell organelles, especially in leaf tissue. The increase in enhanced chlorophyll content in bacteria-treated plants under salt stress on Petri plates signified that inoculation with the PGPR identified in the current work counteracted the negative effects of salinity stress on photosynthetic activity, resulting in a positive effect on growth and plant development. This might be partially due to bacterial ACCD (1-aminocyclopropane-1-carboxylate (ACC) deaminase) activity^66^ or induced systemic resistance in plants, both of which would have led to reduced ethylene biosynthesis. It should be noted that the total chlorophyll content was not significantly changed in treated *A. thaliana* plants in the pot experiment, which was not in accordance with previous results from the in vitro experiment, suggesting that the pattern of beneficial effects exerted by rhizobacteria is at least partially affected by growth conditions and salt stress levels.

Some PGPR were reported to promote plant growth under both normal and stressed conditions^67,68^, while others were effective only under stressed conditions, exerting no growth promotion effect under optimal conditions^69^, suggesting that the PGP activity of some rhizobacteria depend on stress^70^. Our results showed the growth stimulating effects of the four tested bacteria existed in soil-free sterile unstressed conditions, suggesting direct effects of these strains, rather than indirect effects on other rhizosphere microbes. Indeed, there have been other reports of growth promotion effects following bacterial inoculation of plants under optimal conditions (sterile and full nutrient)^71,72^, thus, the plant growth-promoting effects observed in the present study probably rely more on the production of plant growth regulators (auxins, gibberellins, cytokinins, etc.), either emitted by the bacteria themselves or produced by plants upon biopriming. Moreover, the non-sterile conditions (peat pellets) used in our *in-planta* assay obviously resulted in a complex microbe-interaction network in the rhizosphere^62^, which was more natural than the sterile conditions**.** In the case of long-term growth, the inoculation effects of strains L, K and Y were also positive; the same results have been reported in *A. thaliana* and various crops by PGPR^73^.

We also investigated the fate of bacterial root colonization in *A. thaliana* plantlets by using GFP-tagged derivatives, since efficient root colonization by inoculated bacteria is a critical step in the initiation of beneficial interactions between bacteria and their host plants^74,75^. An endophyte, *Burkholderia phytofirman* PsJN^T^, from onion roots, successfully colonized the roots of various plant species, such as *Arabidopsis*^76^, corn^77^, and switchgrass^78^. Although strains n, L and Y, respectively, were identified as endophytes from surface-sterilized roots of reed canary grass, bittersweet, and soybean, transformed strains colonized the interior part of root tissues of *Arabidopsis* 14 DAS. Uninoculated seedlings and seedlings inoculated with wild-type strains did not show any fluorescence. Results obtained from this study indicated that strains n, L and Y were better colonizers of *Arabidopsis*. Plate counting showed that strain K efficiently colonized root tissue. Endophytic bacteria of the four strains were detected in the range 10^3^–10^6^ CFU mg^−1^ FW (Table S4). GFP has been used to determine the colonization pattern of *Enterobacter* sp.^79^ and *Rhizobium* sp.^80^ in *A. thaliana*, and *Bacillus* sp. in rice^81^ and Chinese cabbage^82^. The present study has shown that the GFP technique is effective in evaluation of colonizing ability of various species of rhizobacteria, although not all species.

Salinity stress imposes oxidative stress and osmotic stress, especially ion (Na^+^ and Cl^−^ ion) toxicity, and reduces plant growth^83^. However, plants alleviate salt stress effects through various mechanisms, such as synthesis of compatible solutes (osmolytes), induction of an antioxidant defense system, and adaptive regulation of stress-related hormones^84^. There was a 2.6-fold increase in proline concentration in plants subjected to salt stress, indicating that proline was accumulated in response to salt stress. There were no differences in proline content between inoculation treatments and controls under normal conditions, which is consistent with the result published by García et al.^85^, indicating that PGPR inoculation did not influence proline content under un-stressed conditions. Inoculation with strains L or Y significantly enhanced the proline content in salt stressed *A. thaliana*, indicating their ability to improve salt tolerance in plants, which correlated well with the phenotypic performance when exposed to salt stress. Proline has been reported to reduce the injury induced by abiotic stresses through several functions, including osmotic adjustment (to maintain turgor pressure), molecular chaperones, signal transduction, and ROS scavenging (to protect cells against oxidative damage)^86^. Under stress conditions, proline itself reacts with and detoxifies ROS; thus, its accumulation may be an important factor to maintain high phtotsynthesic rates^87^. It is plausible that increases in proline content following inoculation under salt stress is employed by strains L and Y to enhance plant growth under salt stress conditions, while for strain K, it is probably the total soluble sugars^56^. Since ROS homeostasis is essential in protecting normal metabolism in plants, alleviation of oxidative damage by ROS-scavenging antioxidant enzymes, such as APX, CAT and POD, is an important stress tolerance enhancement strategy in plants^88^. The CAT catalysis pathway has been considered as a key ancillary component of photosynthesis that can efficiently remove the photorespiratory H_2_O_2_ produced by plants under drought and salinity stress^89^. APX also plays a key role in the conversion of H_2_O_2_ into H_2_O. It is of particular importance in maintaining the homeostasis of two non-enzymatic antioxidants, ascorbate and glutathione, and is involved in the prevention of the over-reduction of photosynthetic rates under stressed conditions^90^. While the DPPH scavenging activity was only significantly increased by stain L inoculation, how the anti-oxidative enzyme activities were regulated in *A. thaliana* exposed to different bacterial isolates under salinity stress is likely to be very complex with metabolic adjustments of the plants response to rhizobacteria under salt stress conditions being dynamic and multifaceted.

The analysis of 16S rRNA gene sequences revealed that strain n is a *Pseudomonas*, L a *Bacillus*, K a *Mucilaginibacter*, and strain Y a *Rhizobium*. Bacterial strain K showed 97% similarity with *M. lappiensis* type strain ANJLI2^T^, which has been reported as a plant-associated bacterium isolated from a decaying lichen in Scots pine forests^91^. This is the first study of the PGP effects of a *Bacillus* from bittersweet, and a PGP strain, *Mucilaginibacter* sp., associated with *Scorzoneroides autumnalis* (wild dandelion)*.*

All-in-all, the four PGPR not only promoted plant growth and efficiently colonize plant root tissues, but also reduced the detrimental effects induced by salt stress. Climate change has already caused significant negative impacts on crop production. These negative effects are largely attributable to drought, flooding, reduced availability of irrigation water, soil salinization and temperature fluctuations. The four PGPR strains of this study impart salt stress tolerance in *A. thaliana*, allowing the plants to grow under stressed conditions. This will be of great potential in sustainable agricultural practices and in developing more climate change resilient crop production practices.

## Conclusions

In this study we analyzed a number of newly isolated rhizobacteria using an easy and labor- and time-saving in vitro screening method using *A. thaliana.* Four bacterial strains termed n, L, K and Y from different species of crop and wild plants stood out as possible PGPR. Sequencing of 16S rRNA genes revealed them to be species of *Pseudomonas*, *Bacillus*, *Mucilaginibacter* and *Rhizobium*, respectively. From the results of the present study, all four endophytic rhizobacteria promoted plant growth and colonized root tissues, while strains L, K and Y resulted in a better response against NaCl stress by effectively inducing the physiological and biochemical responses inthe plants, allowing them to cope with salinity-induced toxicity. The mechanisms by which isolates exert growth promotion effects in *A. thaliana* were also studied. The increased anti-oxidative defense machinery suggested an induction of systemic response in plants. Moreover, these bacteria promoted plant growth in a nonsterile system on various substrates with different nutrient conditions, indicating that these selected rhizobacterial strains are capable of enhancing plant growth and providing salinity protection under a range of conditions. Moreover, GFP-signals confirmed the association of bacteria with plant roots. This is of great importance in case the growth of beneficial strains be compromised by abiotic soil conditions or they outcompeted by other antagonistic bacteria in the soil environment. These four bacterial isolates could be re-isolated from inoculated plants, with a bacterial colonization density within the range typical for endophytic bacteria^92^. Thus, all four strains reported in this study could be defined as endophytes and are promising candidates for development into commercial biofertilizer formulations for use in agricultural crop production. This is the first study regarding the PGP effects of *Bacillus* sp. from bittersweet. To our knowledge, there is no detailed study about PGP effects by *Mucilaginibacter* (stain K) in *A. thaliana*. Only one paper showed that Mucilaginibacter is an important rhizosphere bacterium on wheat^93^. Our direct screening approach revealed this new taxon as a fitness-enhancing rhizobacterium of *A. thaliana* under either optimal, salinity-stressed or semi-natural conditions. As such this is the first comprehensive report about the potential agricultural applications of *Mucilaginibacter* sp. from an uncultivated plant. The use of environmentally friendly, safe agents to increase agricultural production would be ideal, thus it would be beneficial to study the potential of all four isolates to enhance plant growth in crops and field level.

## Supplementary information

Supplementary information
