# Supplementary material for: Plant endophytes promote growth and alleviate salt stress in Arabidopsis thaliana
Source: Sci Rep. 2020 Jul 29;10:12740. doi: 10.1038/s41598-020-69713-5 (PMC7391687; doi:10.1038/s41598-020-69713-5)
Supplement: Supplementary file 1 — Supplementary information [file 41598_2020_69713_MOESM1_ESM.pdf]

article title: Plant endophytes promote growth and alleviate salt stress in *Arabidopsis thaliana*

journal: Scientific Reports

author: Di Fan, Sowmyalakshmi Subramanian, Donald L. Smith*

Supplementary Fig. S1 Effects of seed bacterization on *A. thaliana* seedling growth. Selected strains were grown and diluted with 10 mM MgSO4 to achieve an OD_{600} of 0.8. Surface sterilized seeds of *A. thaliana* were inoculated with the test strains (1 h incubation at room temperature) and deposit on agar plates. Results are expressed as shoot fresh weight relative to untreated controls after a 3-week growing period on vertical agar plates (* $P < 0.05$).

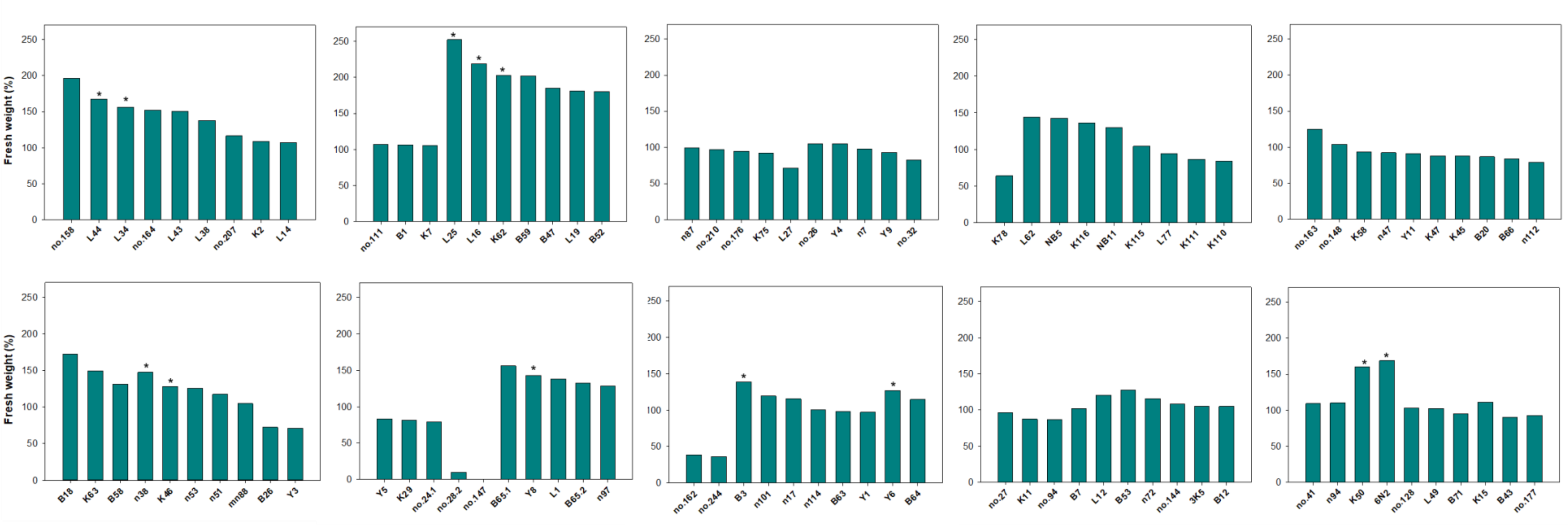

Supplementary Fig. S2 Phylogenetic tree generated using neighbor-joining algorithm based on 16S rRNA gene sequence showing inter-relationship of representative strains with the closely related type strains (accession number in parentheses). The numbers at branch nodes are percentages of bootstrap support based on 1000 resamplings. Bar, 0.01 or 0.05 substitutions per nucleotide position

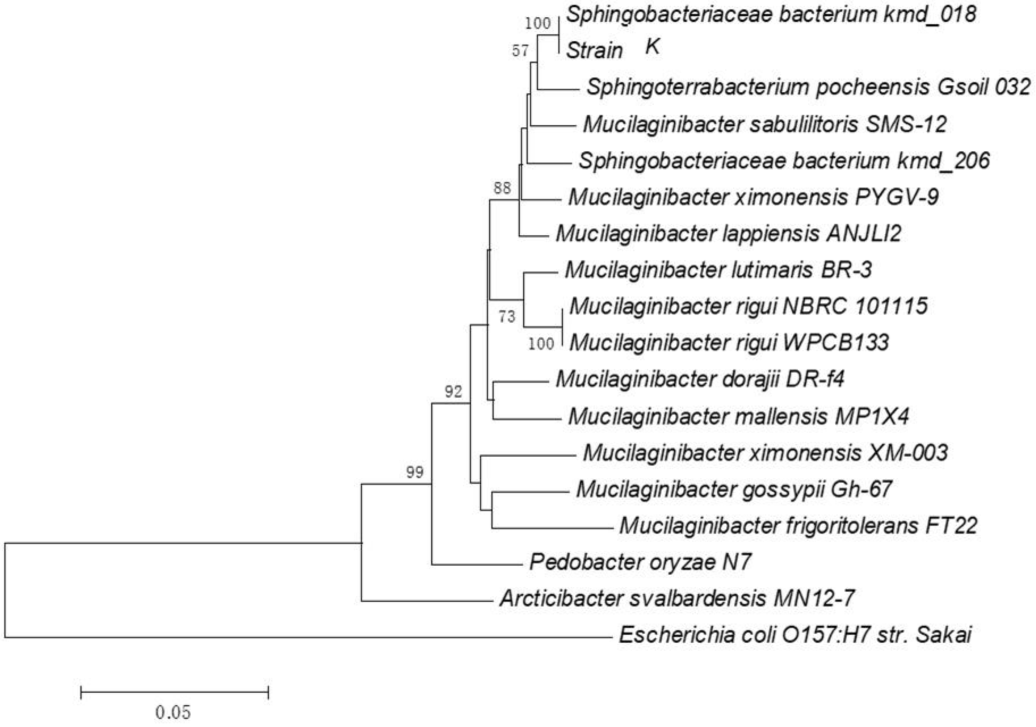

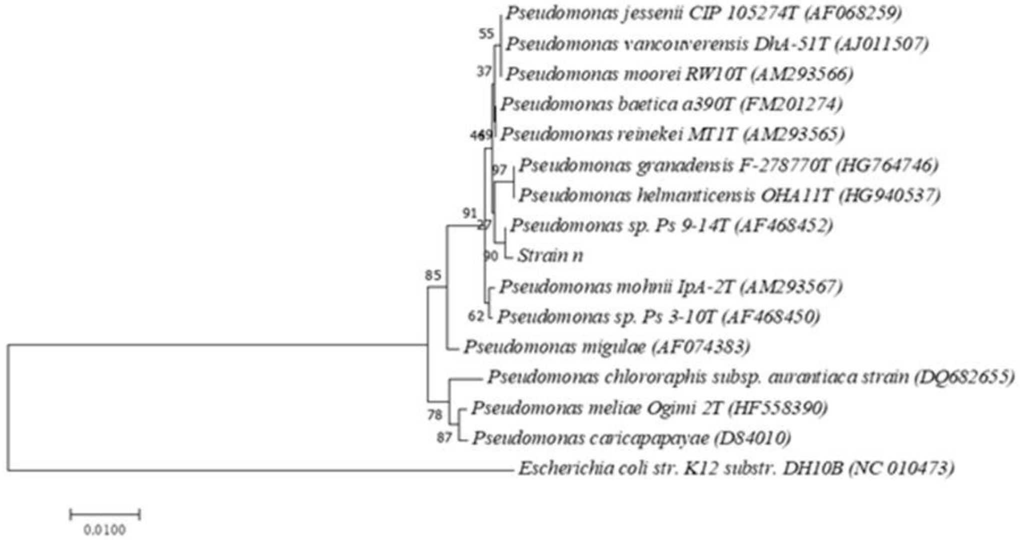

Supplementary Fig. S3 Comparison of germination rate of *A. thaliana* under normal, salt, and osmotic stress conditions. The control and treated seeds were germinated on ½ MS agar plates (a) supplemented with 100 mM (b) and 150 mM (c) of NaCl and mannitol (100 and 300 mM) (d), respectively. The germinated seeds were scored regularly up to 10 d. The results are mean ± standard error from three independent experiments, with 100 seeds per treatment.

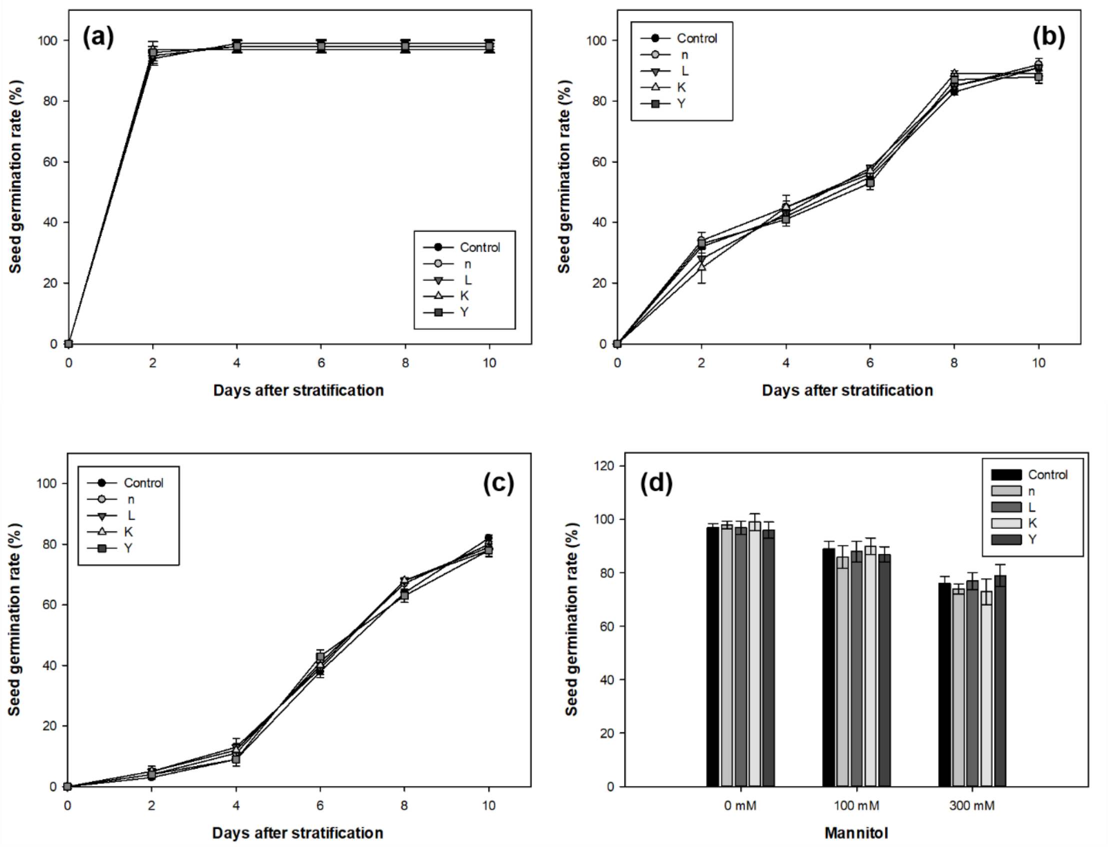

Supplementary Fig. S4 Visualization of rhizobacteria labeled with pDSK-GFPuv plasmid. The 3 isolates (*Pseudomonas* sp. (n), *Bacillus* sp. (L) and *Rhizobium* sp. (Y)) indicated were successfully tagged with GFP (pDSK-GFPuv, KanR) (out of 4 isolates attempted). Transformed bacteria were cultured in liquid KB medium supplemented with kanamycin overnight. Pictures show GFP-labeled bacteria under a fluorescent microscope (b) and GFP-labeled bacteria in white light (a)

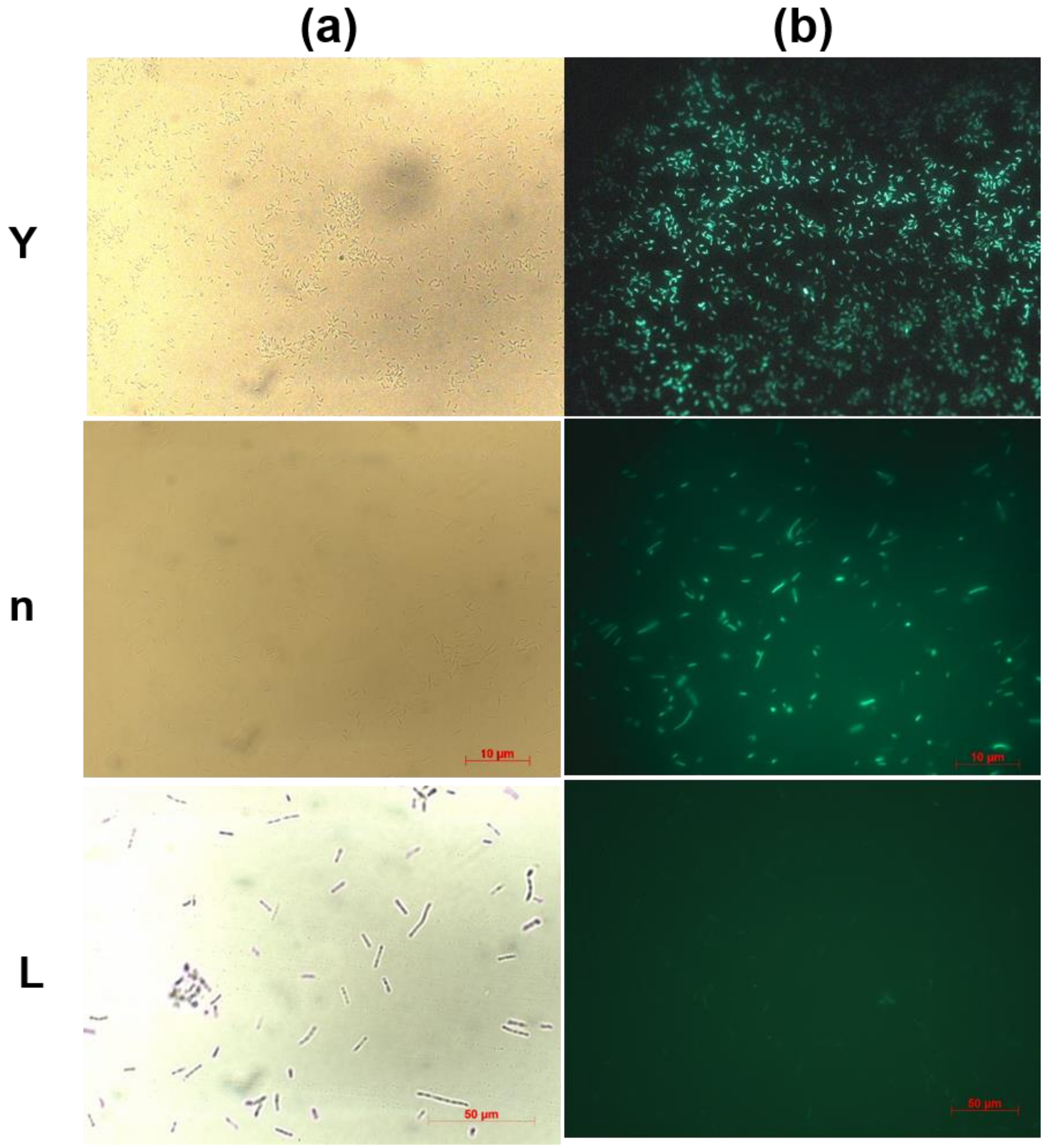

Table S1. The growth of *A. thaliana* plants in sterilized potting soil. Total leaf surface area, and rosette fresh and dry weight were measured after 28 d of co-cultivation with the indicated bacterial treatment.

Strains	Total leaf surface area	rosette fresh weight	rosette dry weight
	percent change to control		
**n**	28	30	25
**L**	52	83	48
**K**	57	76	43
**Y**	48	80	50

Table S2. The growth of *A. thaliana* plants under non-sterile conditions (peat pellets). Total leaf surface area, and rosette fresh and dry weight were measured after 28 d of co-cultivation with the indicated bacterial treatment.

Strains	Total leaf surface area	rosette fresh weight	rosette dry weight
		percent change to control	
**n**	15	11	9
**L**	36	60	33
**K**	40	51	29
**Y**	31	43	27

Table S3. Effect of bacteria inoculation on the activities of APX, CAT and POD in *A. thaliana*. Results are expressed as enzyme activity relative to untreated controls, are given as mean from three independent experiments.

Strains	APX	CAT	POD
	percent change to control		
**n**	1.9	1.1	4.9
**L**	3.6	3.1	1.7
**K**	2.8	2.3	2.4
**Y**	1.1	3.0	1.5

Table S4. Population density (CFU) of the four selected bacterial strains from roots and rosettes of 14-day-old *A. thaliana* seedlings. Data are presented as numbers of CFU mg^{-1} fresh weight.

Treatments	rosettes	roots
**n**	$1.8*10^{4}$	$2.8*10^{6}$
**L**	$1.1*10^{2}$	$1.8*10^{3}$
**K**	$6.3*10^{3}$	$5.9*10^{6}$
**Y**	$8.9*10^{4}$	$4.8*10^{5}$
